# Burden of Breast Cancer and Its Attributable Risk Factors in the Belt and Road Initiative Countries, 1990–2021

**DOI:** 10.1111/1759-7714.70186

**Published:** 2025-11-16

**Authors:** Tiankun Wang, Huimin He, Hao Zi, LiSha Luo, Qiao Huang, Xingpei Guo, Wenting Zhou, Tong Deng

**Affiliations:** ^1^ Center for Evidence‐Based and Translational Medicine Zhongnan Hospital of Wuhan University Wuhan China; ^2^ Department of Thyroid and Breast Surgery Zhongnan Hospital of Wuhan University Wuhan China; ^3^ Department of Gastroenterology People's Hospital of Henan Provincial People's Hospital, School of Clinical Medicine, Henan University Zhengzhou Henan Province China; ^4^ Evidence‐Based Medicine Center Xiangyang No. 1 People's Hospital, Hubei University of Medicine Xiangyang China; ^5^ Department of General Surgery Zhengzhou Central Hospital Affiliated to Zhengzhou University Zhengzhou China; ^6^ Administrative Office of President, Zhongnan Hospital of Wuhan University Wuhan China

**Keywords:** belt and road initiative countries, breast cancer, cancer epidemiology, global burden of disease, risk factor

## Abstract

**Background:**

This study evaluated the burden of breast cancer (BC) in Belt and Road Initiative (BRI) countries from 1990 to 2021 by age, sex, and risk factors, aiming to guide health promotion.

**Methods:**

Data from 66 BRI countries were extracted from the Global Burden of Disease (GBD) 2021 database. Trends in age‐standardized incidence (ASIR) and mortality (ASMR) were assessed using estimated annual percentage change (EAPC). Associations with the Social Development Index (SDI) and attributable risk factors were also examined.

**Results:**

From 1990 to 2021, global ASIR rose from 21.38 to 24.56 per 100 000 (EAPC = 0.38), while ASMR declined by 0.59% annually; BRI countries showed similar trends. In 1990, Greece had the highest ASIR and Israel the highest ASMR, whereas Bangladesh and Oman had the lowest. By 2021, Lebanon reported the highest ASIR, Georgia the highest ASMR, and Mongolia and Oman the lowest. Turkey showed the largest ASIR increase (EAPC = 6.77). Both ASIR and ASMR were positively correlated with SDI. Risk factors also shifted: in 1990, major contributors were high red meat intake, high body mass index (BMI), and alcohol use; by 2021, high red meat intake, high BMI, and high fasting plasma glucose (FPG) dominated. High BMI and FPG rose markedly, whereas smoking, secondhand smoke, and alcohol use declined.

**Conclusion:**

Although mortality has fallen, BC incidence continues to rise in BRI countries, especially in low‐ and middle‐SDI regions. Expanded screening, improved healthcare infrastructure, and targeted interventions on modifiable risks are urgently required.

## Graphical abstract

Breast cancer incidence is rising while mortality declines in BRI countries, with variations by region and SDI. High BMI and fasting plasma glucose are emerging drivers, highlighting the urgent need for improved screening, healthcare infrastructure, and targeted interventions to reduce modifiable risk factors.

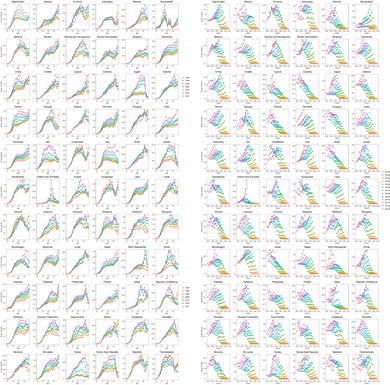

## 1 Introduction

Cancer is one of the leading causes of death worldwide and constitutes a significant public health challenge [1]. Among the various types, breast cancer (BC) has emerged as the most prevalent cancer and is one of the most burdensome diseases globally [2]. In 2022, BC was the second main cause of cancer incidence worldwide, with an estimated 2.3 million new cases, accounting for 11.6% of all cancer cases. The disease is the principal cause of cancer deaths, considering incidence rates in 157 countries and mortality in 112 countries, thus representing a severe health threat and economic burden to individuals, families, and society [1]. Although male BC represents about 1% of all BC globally, its incidence rate appears to be on the rise [3]. Therefore, a comprehensive analysis of the disease burden of BC can offer valuable insights for its comprehensive management.

As international exchanges deepen swiftly, health issues have transcended national boundaries [4]. Research indicates significant geographical disparities in the disease burden of BC, with the highest burden observed in countries undergoing economic transition [5]. The Belt and Road initiative refers to the development project initiated by China to strengthen infrastructure, trade development, and business partnerships among 66 countries across Asia, Europe, South America, and Africa [6]. In 2017, China introduced the “Health Silk Road” initiative to enhance global health cooperation [7]. Against the backdrop of COVID‐19, the Belt and Road initiative provides an important platform for member countries to discuss clinical treatment guidelines and epidemic control strategies. It also offers an opportunity to promote international cooperation on health issues, including the fight against BC [8]. However, current research lacks a consistent assessment of the BC burden in Belt and Road Initiative (BRI) countries and has not thoroughly revealed the epidemiological variations of BC along with its risk factors and their links to age and socio‐economic status. Hence, there is a critical need for relevant studies to address the rising disease burden and regional disparities of BC.

The World Health Organization's Global Breast Cancer Initiative was established in 2021 with the aim of reducing BC mortality, envisioning saving millions of lives through various approaches emphasizing health promotion, early detection, timely diagnosis, and comprehensive management [9]. Factors such as alcohol consumption, diet, physical activity, smoking habits, body mass index (BMI), and blood glucose levels significantly influence the development of BC. Therefore, managing these risk factors is crucial for preventing BC onset.

This study aims to address this gap by conducting a comparative analysis of the incidence and mortality rate trends of BC globally, and specifically within BRI countries. Utilizing data from the GBD 2021, this research aims to dissect the dynamics of BC within different age groups. Additionally, the study explores how the Socio‐Demographic Index (SDI) and risk factors correlate with health outcomes, elucidating the contributory factors to these associations. The outcomes are expected to offer a comprehensive comparative perspective on the BC burden in the BRI region, thereby informing the distribution of medical resources and assisting policymakers in devising tailored prevention and control strategies.

## 2 Materials and Methods

### 2.1 Data Source

This study is based on GBD 2021 data, which encompasses health detriments related to 369 diseases, injuries, and 88 risk factors for 204 countries and territories [10]. Information on BC was collected from the Global Health Data Exchange (GHDx) online data retrieval tool (http://ghdx.healthdata.org/gbd‐results‐tool). A comprehensive description of the methodology adopted in this study has been previously published [2, 10]. This study extracted data on incidence, mortality, Disability‐Adjusted Life Years (DALYs), and their respective age‐standardized rates (ASR) from the GBD 2021 database, disaggregated by sex, age, and specific risk factors for the years 1990 to 2021, for countries along the Belt and Road. Additionally, the study employed the SDI, an indicator that quantifies socio‐demographic progress in a region based on income, education, and fertility conditions [11].

### 2.2 BRI Countries

The BRI countries include 66 member countries, divided as follows: (1) East Asia: China; (2) Central Asia: Armenia, Azerbaijan, Georgia, Kazakhstan, Kyrgyzstan, Mongolia, Tajikistan, Turkmenistan, and Uzbekistan; (3) South Asia: Bangladesh, Bhutan, India, Nepal, and Pakistan; (4) Southeast Asia: Cambodia, Indonesia, Laos, Malaysia, Maldives, Burma, the Philippines, Sri Lanka, Thailand, and Vietnam; (5) high‐income Asia Pacific: Brunei and Singapore; (6) North Africa and the Middle East: Afghanistan, Bahrain, Egypt, Iran, Iraq, Jordan, Kuwait, Lebanon, Oman, Palestine, Qatar, Saudi Arabia, Syria, Turkey, the United Arab Emirates, and Yemen; (7) Central Europe: Albania, Bosnia and Herzegovina, Bulgaria, Croatia, Czechia, Hungary, Montenegro, North Macedonia, Poland, Romania, Serbia, Slovakia, and Slovenia; (8) Eastern Europe: Belarus, Estonia, Latvia, Lithuania, Republic of Moldova, Russia, and Ukraine; (9) Western Europe: Cyprus, Greece, and Israel [12].

### 2.3 Attributable Risk Factors

The GBD classifies Level 1 risk factors into behavioral, metabolic, and environmental/occupational risks. Attributable risk factors for breast cancer include seven risks: alcohol consumption, diets high in red meat, smoking, secondhand smoke, and low physical activity as behavioral risks, and high BMI and high fasting plasma glucose (FPG) as metabolic risks [13]. This study analyzed the burden of breast cancer and trends in these behavioral and metabolic risks.

### 2.4 Socio‐Demographic Index

SDI is a composite measure that reflects the overall developmental status of a country. It quantifies socio‐demographic progress based on income, education, and fertility rates [14]. The SDI ranges from 0 to 1, divided into five levels: high (0.805129–1), upper‐middle (0.689504–0.805129), middle (0.607679–0.689504), lower‐middle (0.454743–0.607679), and low (0–0.454743) [15].

### 2.5 Statistical Analysis

The estimation process of BC has been introduced in GBD 2021 publications. The 95% uncertainty interval (UI) were reported for all estimates. The ASR (per 100 000 population) was calculated by the sum of the products of age‐specific rates (*a*_*i*_, where *i* denotes the *i*th age) and the population number (or weight *w*_*i*_) in the same age group i of the selected reference standard population, divided by the sum of the standard population weights: ASR = ASR = $\frac{\sum_{i=1}^{A}a_{i}w_{i}}{\sum_{i=1}^{A}w_{i}}$ × 100 000. The estimated annual percentage changes (EAPC) were used to describe the trend of ASR within a specified time interval. The EAPC were estimated by a linear regression model: *y* = *α* + *βx* + *ϵ*, where *y* is ln(ASR), *x* is the calendar year, and *ε* is the error term. The EAPC were calculated as 100 × (exp(*β*) − 1) and its 95% confidence interval (CI) can be obtained from the linear regression model [16, 17]. When the estimated EAPC value and its lower 95% CI were both > 0, ASR is considered as an upward trend. Conversely, if the estimated EAPC value and its upper 95% CI were both < 0, ASR is considered a downward trend. The relationship between BC incidence and mortality and three main effects (age, period, and cohort) were investigated using the BRI country‐period‐cohort framework. Data on BC outcomes and populations were divided into 5‐year intervals of age (20–84 years), period (1990–2019), and birth cohort (period‐age) to fit the models. The detailed methods are described in previous publications [18]. R software (Version 4.2.3) and Microsoft Excel (Version 2019) were used for statistical analysis and visualization.

## 3 Results

Table 1 shows the ASIR and ASMR for BC globally, in SDI regions, and BRI countries for the years 1990 and 2021. In 2021, the global ASIR was 24.56 per 100 000 persons (95% UI: 22.93–26.26), compared to 21.38 per 100 000 persons (20.27–22.22) in 1990, showing an increasing trend (EAPC% = 0.38; 95% CI: 0.24–0.71). Conversely, between 1990 and 2021, the global mortality rate for BC showed a downward trend, decreasing by 0.59 annually (EAPC%; −0.64 to −0.54).

**TABLE 1:** The age‐standardized incidence, mortality rates in 1990 and 2021, and their corresponding EAPC of breast cancer among RBI countries and globally.

Characteristics	Age‐standardized incidence rate (per 100 000) (95% UI)		EAPC (95% CI) 1990–2021	Age‐standardized mortality rate (per 100 000) (95% UI)		EAPC (95% CI) 1990–2021
	1990	2021		1990	2021	
Global	21.38 (20.27 to 22.22)	24.56 (22.93 to 26.26)	0.38 (0.33 to 0.42)	9.16 (8.57 to 9.61)	7.90 (7.27 to 8.44)	−0.59 (−0.64 to −0.54)
Sex						
Female	39.99 (38.01 to 41.60)	46.40 (43.26 to 49.56)	0.4 (0.35 to 0.45)	16.60 (15.60 to 17.45)	14.55 (13.45 to 15.56)	−0.36 (−0.52 to −0.20)
Male	0.52 (0.46 to 0.60)	0.94 (0.60 to 1.15)	2.22 (2.06 to 2.38)	0.28 (0.24 to 0.34)	0.34 (0.23 to 0.41)	0.45 (0.25 to 0.65)
Socio‐demographic index						
Low SDI	8.23 (7.08 to 9.47)	12.84 (11.35 to 14.30)	1.39 (1.25 to 1.54)	6.53 (5.64 to 7.50)	8.66 (7.66 to 9.66)	0.88 (0.76 to 1.00)
Low‐middle SDI	7.39 (6.64 to 8.31)	14.78 (13.36 to 16.10)	2.27 (2.23 to 2.31)	5.11 (4.55 to 5.76)	7.75 (6.96 to 8.50)	1.37 (1.32 to 1.41)
Middle SDI	10.52 (9.70 to 11.49)	19.54 (17.67 to 21.62)	1.94 (1.90 to 1.99)	5.99 (5.53 to 6.53)	6.79 (6.16 to 7.54)	0.28 (0.23 to 0.33)
Middle‐High SDI	21.19 (20.14 to 22.16)	27.24 (24.69 to 30.42)	0.76 (0.69 to 0.83)	9.70 (9.14 to 10.17)	7.68 (6.98 to 8.37)	−0.92 (−1.02 to −0.82)
High SDI	43.25 (41.36 to 44.25)	40.17 (37.29 to 41.71)	−0.27 (−0.39 to −0.16)	13.44 (12.69 to 13.87)	8.38 (7.55 to 8.82)	−1.62 (−1.66 to −1.57)
East Asia						
China	8.54 (7.07 to 10.10)	18.32 (14.50 to 22.93)	2.50 (2.42 to 2.58)	4.74 (3.96 to 5.57)	4.85 (3.91 to 5.92)	−0.50 (−0.62 to −0.39)
Central Asia						
Armenia	31.43 (29.48 to 33.57)	27.56 (23.73 to 31.58)	−0.43 (−0.74 to −0.11)	16.57 (15.64 to 17.48)	11.65 (10.20 to 13.50)	−1.06 (−1.36 to −0.75)
Azerbaijan	17.55 (15.85 to 19.45)	17.38 (12.88 to 22.44)	0.27 (0.05 to 0.49)	10.48 (9.50 to 11.55)	7.78 (5.97 to 9.81)	−0.62 (−0.76 to −0.48)
Georgia	38.21 (34.47 to 42.06)	35.98 (30.90 to 41.83)	0.40 (0.12 to 0.69)	17.93 (16.32 to 19.57)	16.40 (14.12 to 18.94)	0.56 (0.27 to 0.86)
Kazakhstan	19.80 (18.37 to 21.22)	18.15 (15.20 to 21.28)	−0.01 (−0.23 to 0.22)	11.03 (10.33 to 11.76)	7.56 (6.28 to 8.79)	−1.04 (−1.42 to −0.67)
Kyrgyzstan	17.78 (16.51 to 19.25)	14.34 (11.79 to 17.31)	−0.73 (−0.99 to −0.46)	10.60 (9.93 to 11.44)	6.42 (5.29 to 7.67)	−1.56 (−1.75 to −1.37)
Mongolia	6.42 (4.86 to 8.28)	6.30 (4.84 to 7.79)	1.26 (1.14 to 1.37)	4.57 (3.47 to 5.85)	3.28 (2.51 to 4.06)	0.11 (−0.06 to 0.28)
Tajikistan	12.89 (11.41 to 14.49)	10.16 (6.44 to 14.92)	−0.82 (−0.97 to −0.66)	8.06 (7.19 to 8.99)	5.44 (3.61 to 7.71)	−1.22 (−1.34 to −1.10)
Turkmenistan	12.47 (11.62 to 13.38)	14.54 (10.77 to 19.50)	0.91 (0.43 to 1.39)	7.52 (7.08 to 7.98)	6.75 (5.07 to 8.94)	0.01 (−0.44 to 0.46)
Uzbekistan	13.38 (12.48 to 14.31)	13.18 (10.82 to 15.82)	0.16 (−0.08 to 0.39)	7.75 (7.29 to 8.21)	6.16 (5.14 to 7.38)	−0.53 (−0.74 to −0.32)
South Asia						
Bangladesh	8.83 (6.72 to 11.26)	8.36 (6.27 to 10.74)	2.25 (2.07 to 2.43)	6.92 (5.26 to 8.74)	8.36 (6.27 to 10.74)	0.79 (0.64 to 0.93)
Bhutan	7.59 (5.07 to 10.7)	7.72 (5.43 to 10.78)	1.43 (1.29 to 1.58)	6.16 (4.14 to 8.59)	7.72 (5.43 to 10.78)	0.38 (0.27 to 0.50)
India	7.05 (5.79 to 8.29)	12.15 (10.30 to 14.38)	2.38 (2.19 to 2.57)	5.56 (4.6 to 6.47)	12.15 (10.30 to 14.38)	1.44 (1.29 to 1.59)
Nepal	8.38 (5.58 to 11.70)	8.37 (6.04 to 11.46)	1.78 (1.51 to 2.04)	6.75 (4.41 to 9.34)	8.37 (6.04 to 11.46)	0.86 (0.59 to 1.12)
Pakistan	20.26 (14.64 to 27.68)	22.64 (16.37 to 29.83)	1.58 (1.41 to 1.76)	16.19 (11.69 to 22.13)	22.64 (16.37 to 29.83)	1.11 (0.90 to 1.33)
Southeast Asia						
Cambodia	8.19 (6.21 to 10.69)	18.98 (13.71 to 25.17)	2.18 (2.14 to 2.22)	6.33 (4.77 to 8.16)	11.53 (8.37 to 15.07)	1.31 (1.26 to 1.36)
Indonesia	13.08 (11.23 to 15.39)	17.39 (11.69 to 24.75)	1.62 (1.53 to 1.71)	9.08 (7.78 to 10.69)	9.94 (6.55 to 14.24)	0.96 (0.85 to 1.08)
Lao People's Democratic Republic	13.47 (9.06 to 19.97)	14.62 (10.42 to 20.06)	1.65 (1.62 to 1.69)	11.04 (7.61 to 16.08)	9.43 (6.82 to 12.91)	0.87 (0.82 to 0.92)
Malaysia	17.34 (15.34 to 19.74)	30.28 (25.48 to 35.84)	2.01 (1.91 to 2.11)	11.20 (9.90 to 12.83)	13.23 (11.27 to 15.39)	0.80 (0.71 to 0.89)
Maldives	11.92 (7.15 to 17.65)	8.90 (6.92 to 11.22)	1.16 (0.75 to 1.57)	7.97 (5.00 to 11.44)	3.83 (3.03 to 4.79)	−0.54 (−0.84 to −0.25)
Myanmar	24.79 (17.6 to 34.06)	17.62 (13.62 to 23.13)	1.02 (0.94 to 1.09)	18.93 (13.93 to 25.42)	10.21 (7.94 to 13.36)	0.23 (0.14 to 0.33)
Philippines	18.32 (15.95 to 20.76)	22.65 (17.93 to 27.99)	1.26 (1.15 to 1.37)	12.75 (11.13 to 14.27)	12.86 (10.24 to 15.85)	0.77 (0.68 to 0.86)
Sri Lanka	7.61 (6.63 to 8.65)	16.64 (10.74 to 22.81)	2.44 (2.27 to 2.62)	4.81 (4.18 to 5.46)	6.69 (4.41 to 8.98)	0.88 (0.72 to 1.04)
Thailand	10.17 (8.78 to 11.74)	24.43 (18.32 to 31.69)	2.85 (2.59 to 3.12)	6.31 (5.52 to 7.23)	8.62 (6.48 to 10.90)	1.31 (1.08 to 1.54)
Viet Nam	15.09 (11.76 to 18.72)	14.55 (11.00 to 19.28)	2.17 (2.13 to 2.21)	10.69 (8.56 to 13.07)	6.23 (4.74 to 8.08)	0.83 (0.79 to 0.86)
High‐income Asia pacific						
Brunei Darussalam	20.89 (17.09 to 25.53)	24.43 (18.96 to 30.30)	2.01 (1.84 to 2.18)	10.00 (8.39 to 11.97)	9.33 (7.32 to 11.42)	1.04 (0.82 to 1.27)
Singapore	23.60 (21.84 to 25.65)	28.35 (26.07 to 30.91)	1.28 (1.03 to 1.52)	8.44 (8.93 to 7.96)	5.60 (5.04 to 6.05)	−0.91 (−1.07 to −0.75)
North Africa and Middle East						
Afghanistan	9.03 (7.09 to 11.47)	14.29 (7.74 to 24.59)	2.37 (2.21 to 2.52)	7.31 (5.83 to 9.19)	7.27 (4.19 to 11.86)	1.54 (1.45 to 1.63)
Bahrain	19.82 (16.86 to 23.23)	44.81 (34.69 to 57.52)	1.69 (1.45 to 1.94)	12.48 (10.73 to 14.45)	10.64 (8.35 to 13.71)	−0.53 (−0.75 to −0.31)
Egypt	7.08 (6.48 to 7.72)	27.30 (21.98 to 33.75)	4.13 (3.74 to 4.53)	4.78 (4.40 to 5.18)	8.77 (7.14 to 10.67)	2.87 (2.44 to 3.30)
Iran (Islamic Republic of)	9.25 (7.54 to 11.83)	26.46 (23.70 to 29.63)	3.61 (3.30 to 3.92)	5.14 (4.10 to 6.74)	4.75 (4.28 to 5.26)	1.73 (1.45 to 2.02)
Iraq	14.60 (10.60 to 20.04)	31.20 (21.84 to 42.15)	2.87 (2.76 to 2.99)	8.85 (6.42 to 12.10)	8.43 (6.03 to 11.14)	0.98 (0.91 to 1.05)
Jordan	17.69 (13.69 to 22.13)	32.60 (23.20 to 43.77)	2.48 (2.05 to 2.92)	10.49 (8.05 to 13.29)	7.53 (5.40 to 10.05)	0.30 (−0.06 to 0.65)
Kuwait	15.53 (14.09 to 17.06)	26.35 (22.21 to 31.05)	1.55 (1.07 to 2.03)	7.07 (6.41 to 7.71)	4.39 (3.69 to 5.08)	−0.27 (−0.66 to 0.13)
Lebanon	25.33 (20.09 to 31.40)	52.72 (41.84 to 65.00)	2.97 (2.65 to 3.29)	13.74 (11.15 to 17.03)	11.11 (8.88 to 13.76)	0.46 (0.26 to 0.67)
Oman	9.31 (6.63 to 13.33)	9.09 (6.96 to 11.58)	2.68 (2.27 to 3.09)	5.76 (4.11 to 8.21)	2.16 (1.71 to 2.68)	0.60 (0.28 to 0.92)
Palestine	18.40 (12.87 to 26.25)	39.19 (31.64 to 47.83)	2.12 (2.01 to 2.24)	10.65 (7.57 to 15.28)	11.19 (8.99 to 13.56)	0.67 (0.53 to 0.81)
Qatar	16.79 (12.55 to 22.51)	42.59 (31.94 to 56.60)	2.56 (2.28 to 2.83)	10.83 (7.94 to 14.71)	8.44 (6.33 to 11.15)	−0.03 (−0.37 to 0.32)
Saudi Arabia	6.12 (4.47 to 8.26)	18.43 (13.82 to 24.78)	3.67 (3.39 to 3.94)	4.50 (3.27 to 6.22)	4.05 (3.08 to 5.35)	1.09 (0.78 to 1.40)
Syrian Arab Republic	6.64 (4.78 to 8.80)	22.84 (16.73 to 30.62)	2.68 (2.58 to 2.79)	4.01 (2.89 to 5.32)	5.46 (4.01 to 7.26)	0.62 (0.48 to 0.76)
Turkey	10.16 (8.05 to 12.86)	29.92 (23.33 to 36.97)	6.77 (5.92 to 7.63)	6.54 (5.23 to 8.27)	6.81 (5.32 to 8.45)	3.97 (3.24 to 4.71)
United Arab Emirates	13.98 (10.05 to 19.63)	29.08 (21.35 to 38.63)	3.32 (2.77 to 3.87)	9.39 (6.76 to 13.28)	8.90 (6.53 to 11.70)	2.03 (1.41 to 2.65)
Yemen	6.61 (4.06 to 10.88)	9.20 (6.40 to 12.82)	2.92 (2.77 to 3.07)	4.91 (3.03 to 8.14)	3.94 (2.80 to 5.46)	1.70 (1.58 to 1.83)
Central Europe						
Albania	9.51 (8.71 to 10.38)	16.26 (11.60 to 21.82)	2.64 (2.31 to 2.97)	5.14 (4.72 to 5.59)	5.69 (3.98 to 7.62)	0.77 (0.59 to 0.95)
Bosnia and Herzegovina	14.46 (13.28 to 15.89)	25.73 (19.82 to 32.25)	2.36 (2.05 to 2.66)	7.69 (7.10 to 8.38)	9.49 (7.49 to 11.72)	1.14 (0.99 to 1.28)
Bulgaria	28.99 (26.67 to 31.27)	41.37 (33.48 to 49.87)	1.35 (1.23 to 1.47)	10.76 (10.04 to 11.47)	13.19 (10.80 to 15.65)	0.68 (0.55 to 0.82)
Croatia	36.03 (32.90 to 39.34)	36.09 (30.17 to 41.78)	0.36 (0.17 to 0.55)	14.73 (13.59 to 15.89)	10.80 (9.22 to 12.38)	−0.81 (−1.00 to −0.63)
Czechia	33.59 (31.79 to 35.61)	31.53 (26.09 to 37.43)	−0.45 (−0.75 to −0.14)	14.34 (13.71 to 14.87)	9.27 (7.76 to 10.91)	−1.80 (−1.99 to −1.60)
Hungary	33.73 (31.72 to 35.62)	36.06 (30.21 to 42.31)	−0.08 (−0.32 to 0.17)	15.74 (15.05 to 16.33)	11.62 (9.90 to 13.53)	−1.22 (−1.37 to −1.07)
Montenegro	33.08 (27.26 to 41.33)	45.79 (35.15 to 58.06)	1.32 (1.19 to 1.44)	13.34 (11.07 to 16.34)	14.80 (11.55 to 18.73)	0.63 (0.50 to 0.77)
North Macedonia	24.76 (22.02 to 27.84)	34.97 (26.72 to 45.29)	1.29 (0.98 to 1.60)	12.45 (11.14 to 13.97)	13.93 (10.86 to 17.48)	0.43 (0.17 to 0.70)
Poland	22.70 (21.71 to 23.76)	32.69 (28.63 to 36.36)	1.16 (0.99 to 1.33)	12.58 (12.04 to 12.93)	12.09 (10.68 to 13.44)	−0.23 (−0.36 to −0.10)
Romania	18.27 (17.34 to 19.33)	29.93 (26.01 to 33.96)	1.54 (1.39 to 1.69)	9.56 (9.17 to 9.98)	11.49 (10.03 to 13.03)	0.38 (0.25 to 0.51)
Serbia	28.71 (25.37 to 32.53)	40.40 (30.66 to 51.68)	0.89 (0.74 to 1.04)	14.66 (12.94 to 16.69)	14.63 (11.16 to 18.80)	−0.56 (−0.70 to −0.41)
Slovakia	25.25 (23.56 to 27.18)	33.24 (25.57 to 41.08)	0.89 (0.73 to 1.05)	11.78 (11.07 to 12.52)	11.21 (8.46 to 14.15)	−0.29 (−0.40 to −0.19)
Slovenia	36.42 (27.05 to 47.29)	31.66 (25.78 to 38.08)	−0.07 (−0.31 to 0.18)	15.03 (11.55 to 19.30)	9.14 (7.51 to 10.94)	−1.43 (−1.65 to −1.20)
Eastern Europe						
Belarus	25.26 (23.58 to 26.83)	29.74 (23.19 to 37.38)	−0.02 (−0.26 to 0.22)	10.89 (10.38 to 11.45)	8.73 (6.95 to 10.92)	−1.31 (−1.59 to −1.02)
Estonia	30.75 (28.60 to 32.94)	30.86 (25.16 to 36.51)	0.09 (−0.02 to 0.21)	13.31 (12.52 to 14.08)	9.09 (7.39 to 10.75)	−1.29 (−1.44 to −1.14)
Latvia	28.09 (26.30 to 30.19)	30.54 (24.87 to 36.43)	0.32 (0.13 to 0.51)	12.95 (12.30 to 13.64)	10.84 (8.94 to 12.75)	−0.34 (−0.53 to −0.15)
Lithuania	27.72 (25.86 to 29.74)	30.51 (25.57 to 35.94)	0.21 (0.01 to 0.40)	11.38 (10.81 to 11.97)	10.18 (849 to 1.93)	−0.16 (−0.37 to 0.05)
Republic of Moldova	23.52 (22.08 to 25.06)	27.68 (23.16 to 32.83)	0.57 (0.33 to 0.82)	11.79 (11.19 to 12.41)	10.05 (0.55 to 1.78)	−0.23 (−0.46 to 0.01)
Russian Federation	21.18 (20.61 to 22.10)	33.56 (29.84 to 37.04)	1.09 (0.94 to 1.25)	9.46 (9.17 to 9.80)	10.83 (9.72 to 11.92)	−0.09 (−0.38 to 0.20)
Ukraine	27.17 (25.62 to 28.79)	21.89 (13.88 to 32.23)	−1.27 (−1.45 to −1.08)	14.79 (14.15 to 15.48)	10.18 (6.61 to 14.73)	−1.54 (−1.77 to −1.31)
Western Europe						
Cyprus	33.48 (29.00 to 39.26)	49.42 (40.10 to 59.63)	1.74 (1.38 to 2.10)	13.80 (12.00 to 16.01)	11.39 (9.39 to 13.64)	−0.43 (−0.56 to −0.30)
Greece	38.02 (35.48 to 40.59)	44.28 (40.19 to 48.41)	−0.22 (−0.37 to −0.06)	12.57 (11.92 to 13.11)	11.75 (10.50 to 12.72)	−0.89 (−1.07 to −0.71)
Israel	41.08 (38.18 to 43.84)	36.71 (32.58 to 40.81)	−0.56 (−0.83 to −0.28)	17.38 (16.44 to 18.24)	10.17 (8.73 to 11.23)	−2.02 (−2.24 to −1.79)

Abbreviations: CI, confidence interval; EAPC, estimated annual percentage change; SDI, socio‐demographic index; UI, uncertainty interval.

In different SDI regions, except for high SDI regions, the ASIR showed an increasing trend. The most significant increase was observed in low‐middle SDI regions with an EAPC of 2.27 (95% CI: 1.36–3.10). From 1990 to 2021, changes in ASMR also varied across different SDI regions, with high and upper‐middle SDI regions showing a decreasing trend, whereas low SDI, low‐middle SDI, and middle SDI regions saw an increase in ASMR of BC (Table 1).

Specifically looking at BRI countries, in 1990, Greece had the highest ASIR (43.33/100 000,40.43–46.07), and Israel had the highest ASMR (16.72/100 000, 15.51–17.84); Bangladesh had the lowest ASIR (4.02/100 000, 3.01–5.43), and Oman had the lowest ASMR (1.94/100 000, 1.45–2.61). In 2021, Lebanon had the highest ASIR (52.72/100 000, 41.84–65.01), and Georgia had the highest ASMR (16.40/100 000, 14.12–18.94); Mongolia had the lowest ASIR (6.30/100 000, 4.84–7.79), and Oman remained the country with the lowest ASMR (2.16/100 000, 1.71–2.68) (Table 1).

From 1990 to 2021, almost all BRI countries showed an upward trend in the ASIR of BC (EAPC > 0). The ASIR in most BRI countries has increased relative to the global level, with only 18 countries having ASIR changes less than the global EAPC level. Over the past few decades, about half of the countries have seen an increase in ASMR (EAPC > 0), with Turkey experiencing the most significant increase in ASMR up to 3.97 (EAPC%; 3.24–4.71). However, it is encouraging that 15 countries experienced a greater reduction in ASMR than the global EAPC level (EAPC% = −0.59, −0.6 to −0.54) (Tables 1, S3, and S4).

**FIGURE 1:**
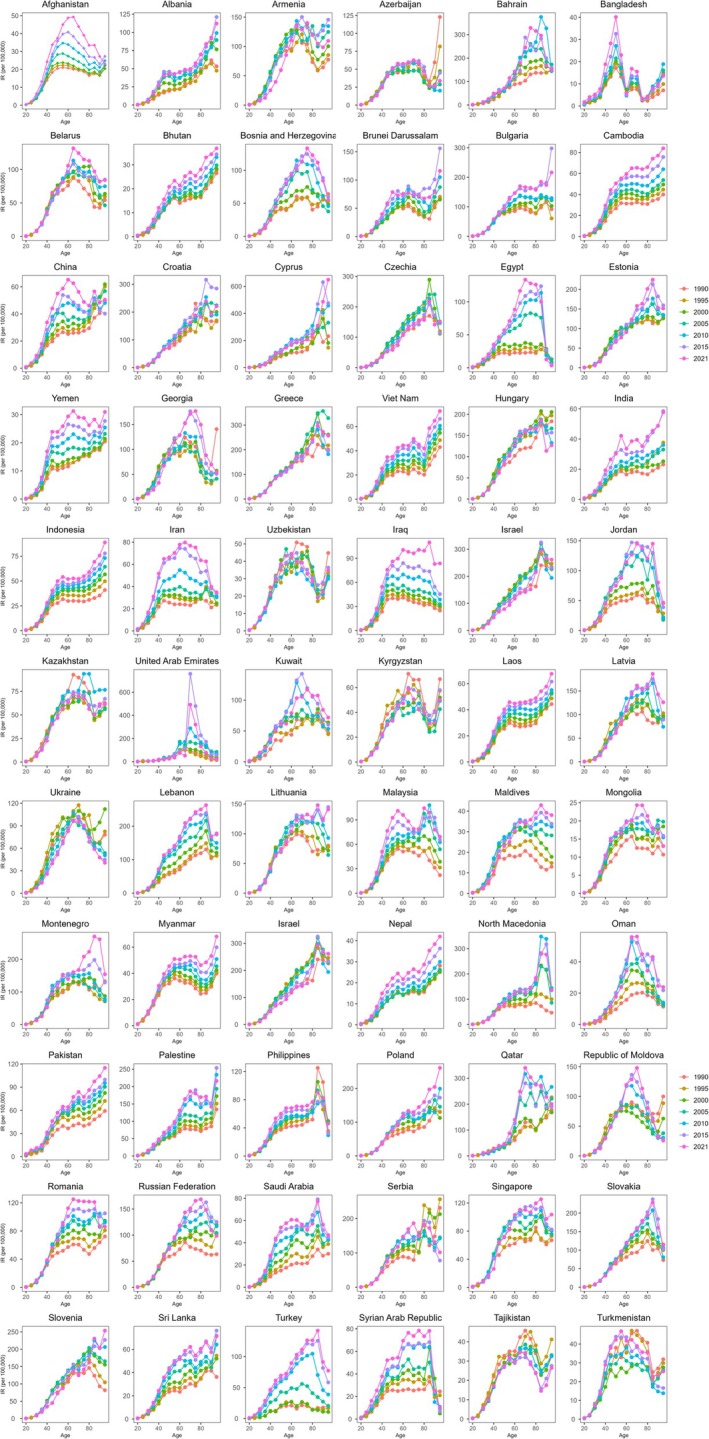
Age‐specific incidence rates of breast cancer by period across 66 BRI countries between 1990 and 2019. IR, incidence rate; BRI, belt and road initiative.

The results indicate that in most BRI countries, the incidence of breast cancer increases with age, peaking around 60 years old before declining. In some countries, such as Uzbekistan, Kyrgyzstan, and Myanmar, the incidence peaks at 60 years old and then increases again at 80. Conversely, in Albania, Cambodia, and Cyprus, the incidence rate continuously increases with age. Generally, the mortality rate of breast cancer increases with age; however, a significant decline around 85 years old is observed in Egypt, Jordan, and the Syrian Arab Republic (Figures 1 and S1).

**FIGURE 2:**
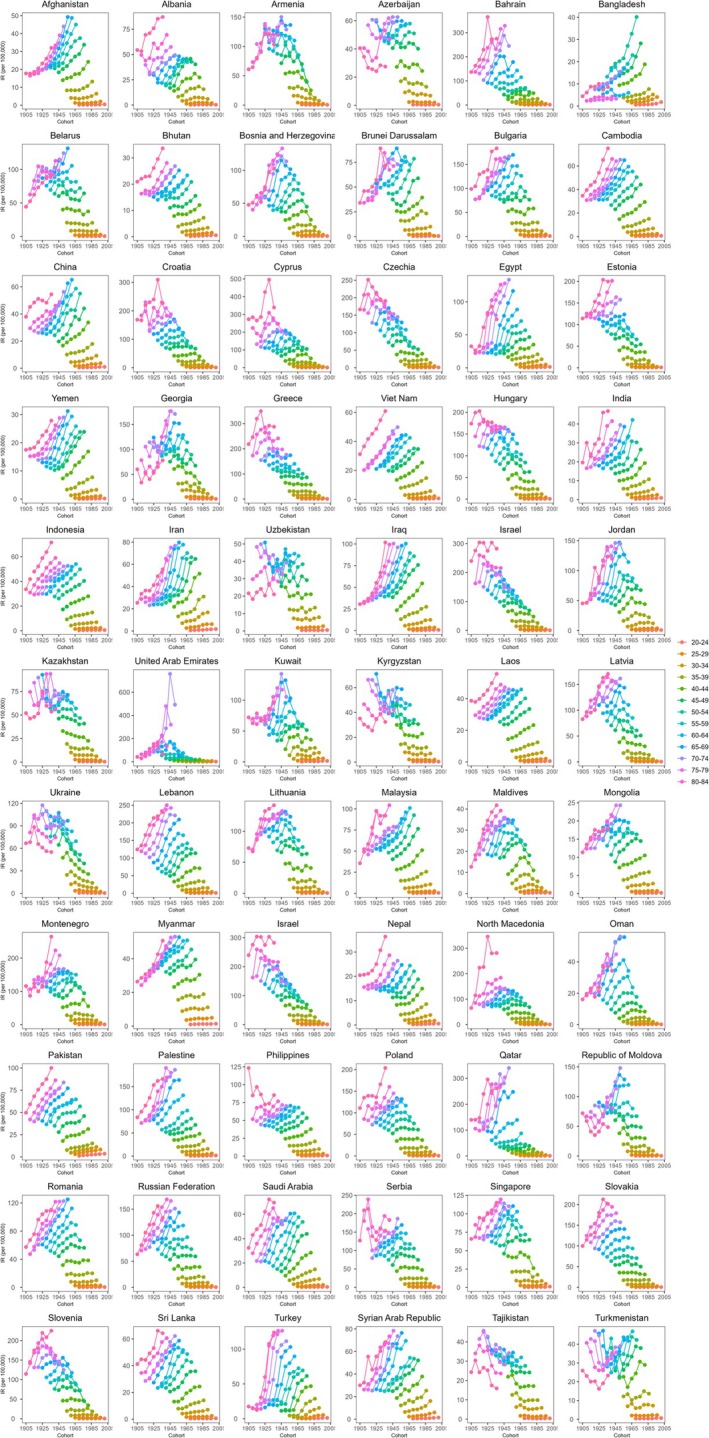
Cohort‐specific incidence rates of breast cancer by age group across 66 BRI countries between 1990 and 2019. IR, incidence rate; BRI, belt and road initiative.

We can see that in the cohort‐related trends of BC incidence and mortality rates by age group, most BRI countries have observed an upward trend in different birth cohorts. But some countries are different, such as Armenia, Greece, Ukraine, and Czechia, where the incidence and mortality rates initially increased, and then decreased in various age groups. There are also some countries where the incidence and mortality rates of birth cohorts first decreased and then increased, such as Azerbaijan, Georgia, the Republic of Moldova and Turkmenistan. The study also found that the incidence and mortality rates of people over 70 years old are always the highest in the birth cohort (Figures 2 and S2).

In 1990, the global ASIR for female was 39.99 per 100 000 (38.01–41.60), and for male, it was 0.52 per 100 000 (0.46–0.60). However, over the past few decades, the ASIR for men has increased at a rate surpassing that of female (Table 1). Regarding the ASMR, the EAPC for female decreased by 0.36% from 1991 to 2021 (−0.5 to −0.20), whereas the ASMR for men has been increasing (0.45%; 0.25–0.65). At a country level, in 2021, the highest ASIR for female was observed in the United Arab Emirates, Qatar, and Bahrain, while the most significant increase in ASIR was seen in Turkey (6.86%, 5.99–7.73). Among males, the highest ASIR was in Lebanon, Croatia, and China, with the most notable upward trend in ASIR observed in Georgia (35.20%, 29.17–41.50). In 1990, the highest ASMR for women was in Israel (30.86/100 000, 28.63–32.95), and for men, it was Lebanon (0.78/100 000, 0.49–1.13). The country with the highest continuous increase in ASMR for women was Turkey, and for men, it was Georgia (Tables S1 and S2).

**FIGURE 3:**
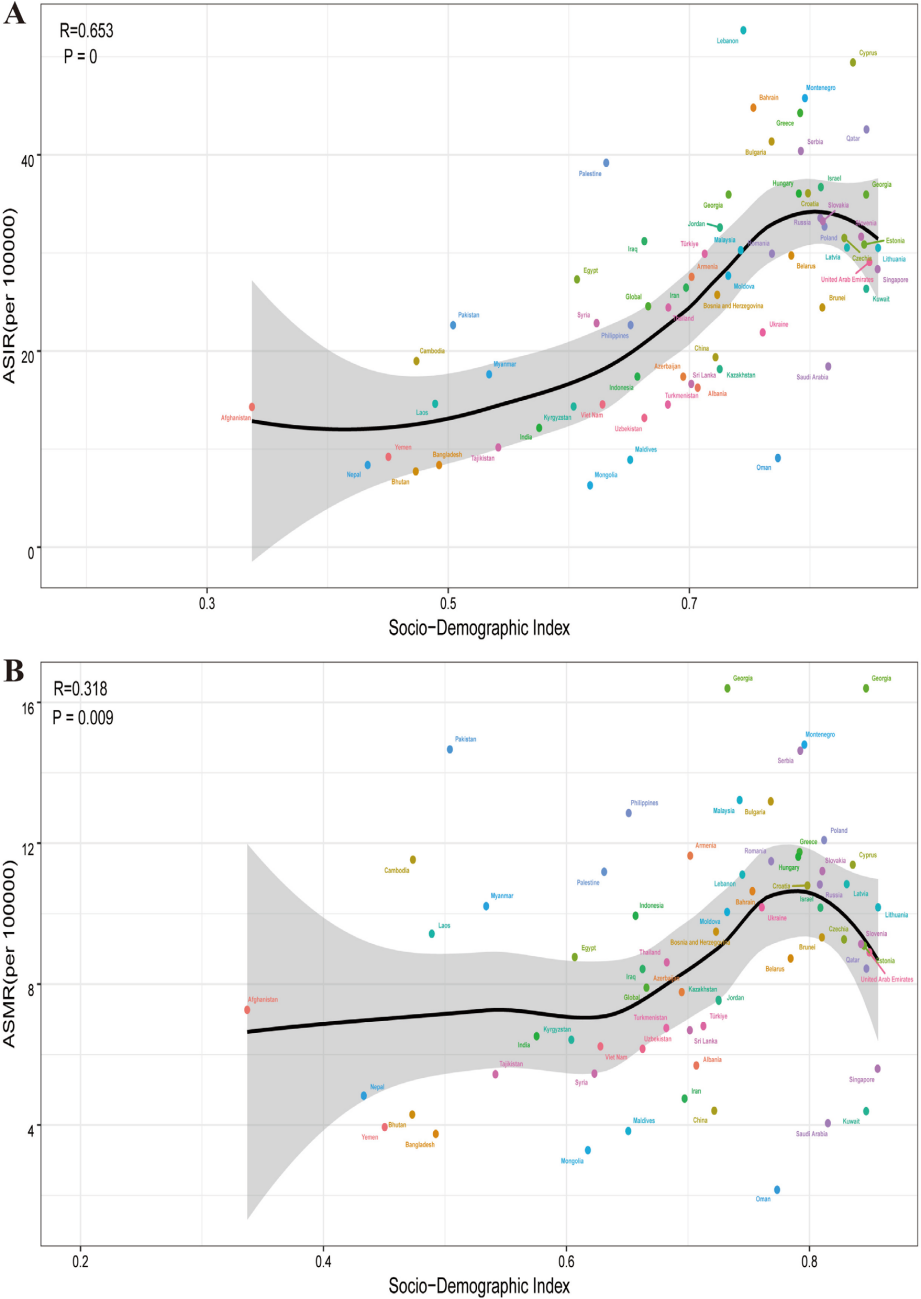
ASIR (A) and ASMR (B) for breast cancer for 66 BRI countries by Socio‐demographic Index, 1990–2021. Each colored point represents the rates for each year from 1990 to 2021 in a specified country. Expected values based on Socio‐demographic Index and disease rates in all locations are shown as the black line. ASIR, age‐standardized incidence rate; ASMR, age‐standardized mortality rate; BRI, belt and road initiative.

The study found a positive correlation between ASIR and SDI of breast cancer in BRI countries in 2021 (*r* = 0.653, *p* < 0.05). ASIR increased with SDI, but this trend changed in countries with SDI greater than 0.8. The relationship between ASMR and SDI is also positively correlated (*r* = 0.318, *p* < 0.05), but when SDI < 0.6, ASMR hardly changes with SDI. When SDI is between 0.6 and 0.8, ASMR increases with SDI. When SDI > 0.8, ASMR decreases (Figure 3).

**FIGURE 4:**
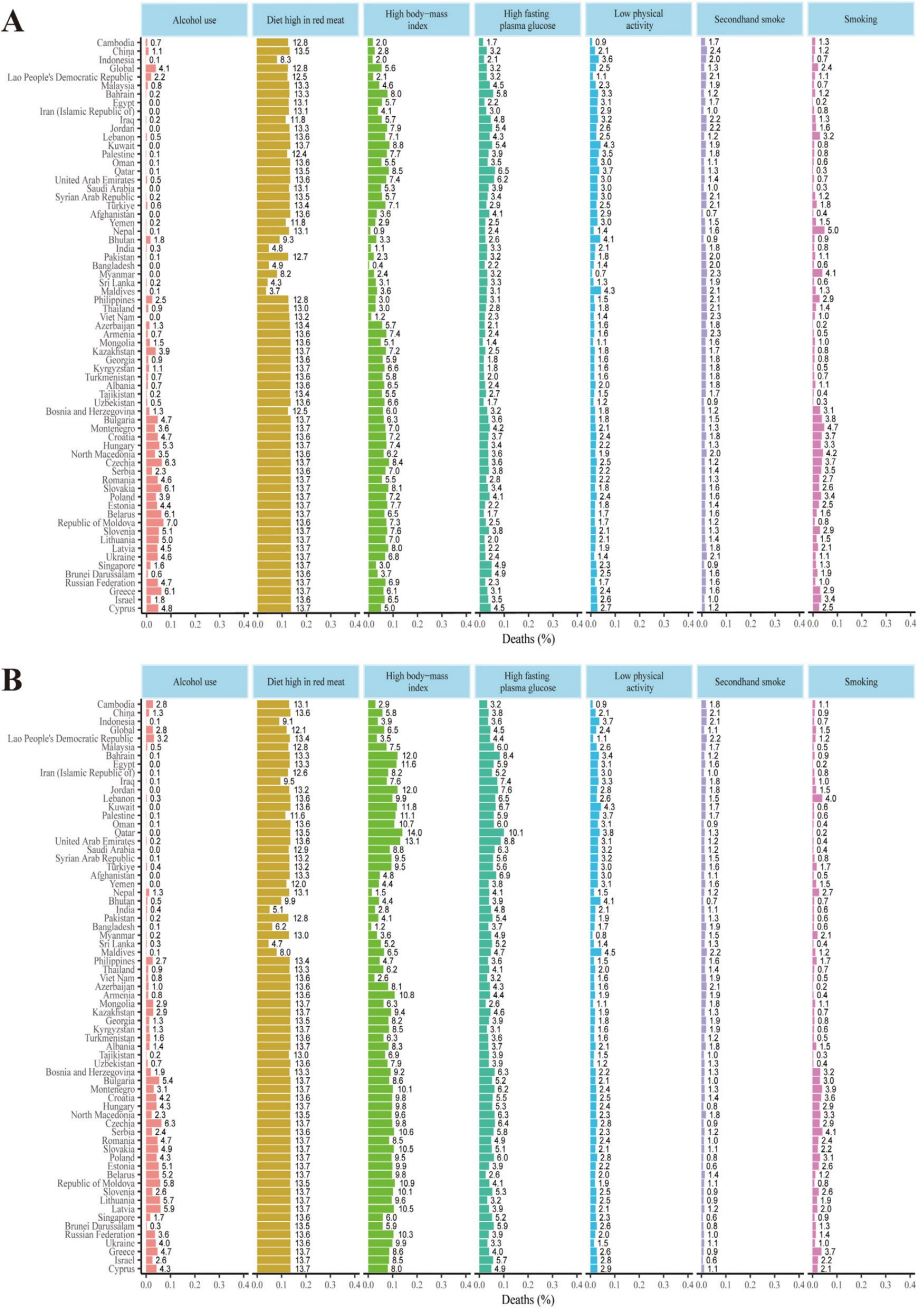
Percentage of Deaths owing to breast cancer attributable to risk factor for 66 BRI countries in 1990 (A) and 2021 (B) in both sexes. BRI, belt and road initiative.

There are seven attributable risk factors for breast cancer globally and in BRI countries: behavioral factors such as alcohol consumption, a diet high in red meat, smoking, secondhand smoke, and physical inactivity, and metabolic factors such as high FPG and BMI. In 1990, the top three factors contributing to breast cancer deaths worldwide were a high red meat diet, high BMI, and alcohol use. By 2021, these top factors had shifted to a high red meat diet, high BMI, and high FPG. The BRI countries mirrored this global trend. The countries with the highest proportion of breast cancer deaths due to a high red meat diet are Greece, Slovenia, and Belarus. For high BMI, the leading countries are Qatar, the United Arab Emirates, and Jordan. Czechia stands out for having the highest proportion of breast cancer deaths caused by secondhand smoke. The countries with the highest breast cancer deaths due to smoking and secondhand smoke are Serbia and the Lao People's Democratic Republic. The Maldives has the highest proportion of breast cancer deaths due to low physical activity. From 1990 to 2021, the most significant increases in risk factors were seen in high BMI and high FPG, while the largest decreases were observed in smoking, secondhand smoke, and alcohol use. The distribution of risk factors for DALYs attributed to breast cancer closely resembles that of deaths (Figures 4 and S3; Tables [Link], [Link]).

## 4 Discussion

BC is the most diagnosed cancer among women and one of the leading causes of cancer‐related deaths worldwide [1]. Despite significant advancements in BC diagnosis and treatment, the rate of development varies across regions and countries, resulting in notable disparities in disease burden [19]. According to the World Health Organization, approximately 70% of cancer deaths occur in low‐ and middle‐income countries [1]. In recent years, higher SDI regions have seen a reduction in disease burden, whereas lower SDI regions have experienced an increase in BC burden, potentially widening these disparities over time. BRI offers a new opportunity to understand BC burden characteristics in developing countries with emerging economies. BRI countries include those from Asia, North Africa, the Middle East, and Europe, each at different stages of economic development. While the BRI primarily focuses on economic development and infrastructure investment, its impact on global health is becoming increasingly apparent [7]. In this study, we used data from the 2021 Global Burden of Disease study to assess the BC burden from 1990 to 2021 in 66 BRI countries, revealing epidemiological trends and regional differences. This analysis provides reliable epidemiological evidence for the Health Silk Road initiative and is the first to report on BC burden among BRI member countries, offering crucial insights for future health policies and resource allocation.

Our study found that from 1990 to 2021, the global ASIR of BC showed an upward trend. Most BRI countries followed this global trend, though the ASIR in high SDI regions worldwide showed a declining trend. Interestingly, in many high SDI BRI countries, the ASIR continued to rise. In higher SDI regions such as High‐income Asia Pacific, Central Europe, Eastern Europe, and Western Europe, the ASIR was higher than the global average, while in low SDI countries, the ASIR was significantly lower. However, the EAPC indicated a decline in incidence burden in high SDI regions. This may be because high SDI countries have long prioritized BC screening, leading to the detection of more early‐stage cases. However, after years of development and effort, the number of new cases has now plateaued [20]. Societal development has led to changes in dietary habits and lifestyles in many developing countries, such as high‐fat diets, high‐sugar diets, lack of exercise, obesity, and alcohol consumption, all of which increase the risk of BC. In BRI countries, most of which are developing nations, economic growth, improved education, and higher living standards have led to changes in reproductive patterns, such as earlier menarche, shorter breastfeeding duration, and later marriage. Additionally, the increasing adoption of Westernized diets and lifestyles, along with a lack of physical activity and sedentary behavior, have also elevated breast cancer risk [21, 22]. With the gradual proliferation of breast cancer screening in Belt and Road countries, the incidence rate of breast cancer is expected to continue rising in the coming years.

Over the past 30 years, the global ASMR for BC has been declining, especially in high SDI regions, where the annual decline is more than twice the global rate. However, in low SDI and middle‐low SDI regions, the ASMR is rising. A similar trend is observed in BRI countries. This is mainly due to advancements in BC treatment methods and the widespread application of mammography screening [23]. Early‐stage BC is considered curable, while metastatic BC has a poor prognosis. Widespread mammography screening has significantly increased the detection of early‐stage BC and reduced deaths from advanced BC [24]. Additionally, improvements in BC treatment, including surgical techniques, radiotherapy, and systemic therapy, have reduced BC mortality. Ongoing research and the development of new treatments, such as cyclin‐dependent kinase 4/6 inhibitors, poly ADP‐ribose polymerase inhibitors, and programmed death‐ligand 1 inhibitors, have greatly improved BC prognosis [25, 26]. However, disparities in development and healthcare levels among countries and regions have led to differences in BC prevention and treatment, which are reflected in the ASMR. In BRI countries like Afghanistan, Iraq, and Yemen, the impact of past wars and conflicts has resulted in a lack of BC screening and care, leading to lower survival rates [27, 28]. In Southeast Asia, countries like the Philippines and Vietnam have not yet implemented BC screening programs and are still in the primary prevention stage (health education), which inevitably leads to higher rates of late‐stage cancer [29]. Our study also found that several South Asian countries have increasing ASMR, which is partly due to economic development and cultural factors. In some Asian countries, women tend to hide their tumors from their families, including their husbands, delaying diagnosis and resulting in poor prognosis [30]. Population aging is another unchangeable risk factor for BC mortality among women in these countries [31].

In terms of gender, BC is the most common type of cancer among women, with high incidence and mortality rates. Although men account for only 1% of total cases, both the ASIR and ASMR for men are rising. In the past 30 years, the global ASIR for men has increased annually by 2.22%, which is approximately five times the growth rate for women. Similarly, the ASMR for men is also on the rise. These findings suggest that male BC should not be overlooked in prevention and treatment strategies. Male patients are usually treated following protocols for postmenopausal women, but they differ from molecular and clinicopathological perspectives [32]. Male BC patients are almost entirely hormone receptor‐positive, including androgen receptors, and are often associated with BRCA2 germline mutations, which significantly increase BC risk [33].

In this study, we analyzed seven risk factors leading to BC deaths and DALYs in BRI countries: behavioral risk factors such as alcohol use, diet high in red meat, smoking, secondhand smoke, and low physical activity, as well as metabolic risk factors like high FPG and BMI. In 2021, behavioral risks were the highest proportion of attributable risk factors for BC deaths in most BRI countries. Notably, a diet high in red meat was the leading risk factor across high, medium, and low SDI countries, and globally. Over the past few decades, dietary patterns have shifted toward high‐energy‐density diets with increased consumption of animal‐derived foods, including red meat [34]. While meat consumption per capita is very high in high‐income countries, it is also growing at an annual rate of 5%–6% in developing countries, with a significant portion being red meat [34]. A meta‐analysis of prospective studies indicated that both unprocessed and processed red meat are associated with an increased risk of B [35]. With economic development, the proportion of red meat consumption has risen significantly in most BRI countries. However, as awareness of the dangers of excessive red meat consumption grows, the proportion of BC deaths attributable to this diet has decreased in nearly half of BRI countries. Compared to 1990, the fastest‐growing attributable risk factors in most countries were high BMI and high FPG in 2021. High BMI and high FPG are also potential risk factors for BC deaths [36]. A prospective chronic disease study found that higher BMI increases the risk of BC for both premenopausal and postmenopausal women [37]. A large European study indicated that both weight and BMI are positively correlated with BC risk [38]. The obesity rate has increased the most in Central Asia, the Middle East, and North Africa, contributing to the incidence and development of BC [39]. High FPG is another important independent risk factor for BC. Women diagnosed with diabetes have a 20%–27% increased risk of BC, possibly due to high FPG accelerating glycation reactions, leading to extracellular matrix stiffening of tumor cells and accelerating BC development [40]. Alcohol consumption is positively associated with overall BC risk, with a stronger association in postmenopausal women compared to premenopausal women [41]. For each additional unit of alcohol consumed per day, the risk of BC increases by 7.1% (95% CI: 5.5–8.7) [42]. Both current and former smokers have a higher risk of BC compared to non‐smokers [43]. However, in most economically advanced BRI countries, the percentage of deaths and DALYs due to these two factors is decreasing. Compared to 1990, although the global burden of BC due to low physical activity has decreased, it is increasing in most BRI countries. Targeting modifiable risk factors is a powerful strategy for preventing disease and injury, leading to poor health and premature death. Our findings are similar to the changes in global or regional trends in breast cancer observed in other GBD studies. However, compared with the world, most countries in the BRI are developing countries, and the risks of BC incidence and mortality remain high [44, 45]. Therefore, strengthening cooperation between regions and countries is beneficial for reducing the burden of BC.

Based on the latest data from the GBD database, this study is the first to reveal the incidence and mortality of BC among BRI countries and to analyze in detail the burden of BC in terms of age, period, sex, and attributable risk factors. For low‐ and medium‐SDI countries, given that resource constraints may limit widespread implementation of mammography, we recommend prioritizing feasible and cost‐effective screening methods, such as clinical breast examinations and breast self‐examination education, as initial measures. We propose multinational collaborative research within the Belt and Road framework to develop and validate cost‐effective, culturally sensitive, and locally appropriate BC screening and prevention strategies. We advocate for the establishment of a formal Belt and Road Breast Cancer Prevention Alliance or similar collaborative platform. Specific, evidence‐based public health campaigns should be implemented targeting the common modifiable risk factors identified in our research. This provides evidence to support future BC prevention and control efforts in these countries. However, our study has limitations. First, although the BC data obtained from the GBD database is rigorously statistically calculated, it may still differ from the actual situation. Second, we focused on ASIR and ASMR without detailed analysis of other indicators. Third, we described the burden of BC at the national level without detailing sub‐national administrative regions. Lastly, we only studied the risk factors included in the GBD database. Despite these limitations, our study is indispensable for formulating clinical guidelines and public health policies for BC.

## 5 Conclusions

This study comprehensively analyzed the burden of BC in BRI countries from 1990 to 2021 and discussed regional and temporal trends. The findings reveal a global upward trend in the ASIR of BC, mirrored in many BRI countries. Notably, high SDI regions showed a decline in BC mortality rates, attributed to effective screening and prevention measures. In contrast, low and middle SDI regions, including several BRI countries, experienced increasing incidence and mortality rates, highlighting the need for improvements in screening and healthcare infrastructure. Economic growth and Westernized lifestyles in BRI countries have increased the risk of BC, with behavioral and metabolic factors such as high red meat intake, obesity, and high FPG playing significant roles. Despite these challenges, the study underscores the importance of international collaboration and targeted interventions to address modifiable risk factors and enhance BC prevention and control efforts.

## Author Contributions

The work reported in the article has been performed by the authors, unless clearly specified in the text. Conceptualization: Wenting Zhou and Tong Deng. Data curation: Tiankun Wang. Formal Analysis: Tiankun Wang, and Huimin He. Investigation: Huimin He. Methodology: Lisha Luo and Qiao Huang. Project administration: Hao Zi and Xingpei Guo. Software: Tiankun Wang. Supervision: Wenting Zhou and Tong Deng. Validation: Hao Zi and Li‐Sha Luo. Writing – original draft: Tiankun Wang and Tong Deng. Writing – review and editing: Tong Deng.

## Ethics Statement

The authors have nothing to report.

## Conflicts of Interest

The authors declare no conflicts of interest.

## Supporting information


**Figure S1:** Age‐specific mortality rates of breast cancer by period across 66 BRI countries between 1990 and 2019. BRI, belt and road initiative; MR, mortality rate.

**Figure S2:** Cohort‐specific mortality rates of breast cancer by age group across 66 BRI countries between 1990 and 2019. BRI, belt and road initiative; MR, mortality rate.

**Figure S3:** Percentage of DALYs owing to breast cancer attributable to risk factor for 66 BRI countries in 1990 (A) and 2021 (B) in both sexes. BRI, belt and road initiative; DALYs, disability‐adjusted life years.

**Table S1:** The age‐standardized incidence, mortality rates and corresponding EAPC of breast cancer for males in BRI countries in 1990 and 2021.

**Table S2:** The age‐standardized incidence, mortality rates and corresponding EAPC of breast cancer for females in BRI countries in 1990 and 2021.

**Table S3:** Percentage of deaths and its change due to breast cancer attributable to risk factors in 1990 and 2021.

**Table S4:** Percentage of DALYs and its change due to breast cancer attributable to risk factors in 1990 and 2021.

**Table S5:** Percentage of deaths and its change due to female breast cancer attributable to risk factors in 1990 and 2021.

**Table S6:** Percentage of deaths and its change due to male breast cancer attributable to risk factors in 1990 and 2021.

**Table S7:** Percentage of DALYs and its change due to female breast cancer attributable to risk factors in 1990 and 2021.

**Table S8:** Percentage of DALYs and its change due to male breast cancer attributable to risk factors in 1990 and 2021.

## Data Availability

Data used for the analyses are publicly available from the Institute of Health Metrics and Evaluation (http://www.healthdata.org/; http://ghdx.healthdata.org/gbd‐results‐tool).
