# Supplementary figures and images for: Burden of Breast Cancer and Its Attributable Risk Factors in the Belt and Road Initiative Countries, 1990–2021
Source: Thorac Cancer. 2025 Nov 16;16(22):e70186. doi: 10.1111/1759-7714.70186 (PMC12620255; doi:10.1111/1759-7714.70186)

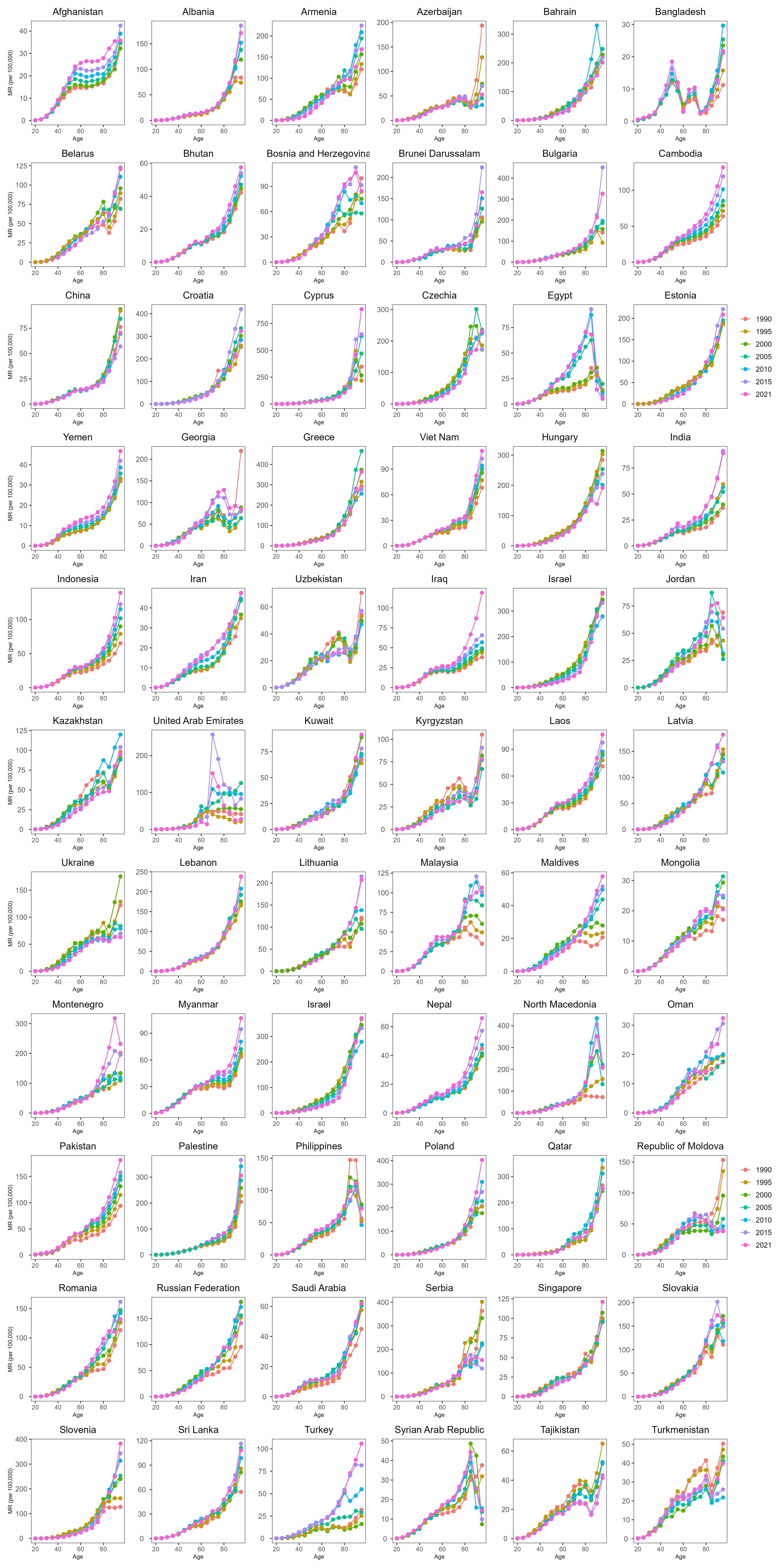

Supplement: Supplementary file 1 — Figure S1: Age‐specific mortality rates of breast cancer by period across 66 BRI countries between 1990 and 2019. BRI, belt and road initiative; MR, mortality rate. [file TCA-16-e70186-s005.tif]

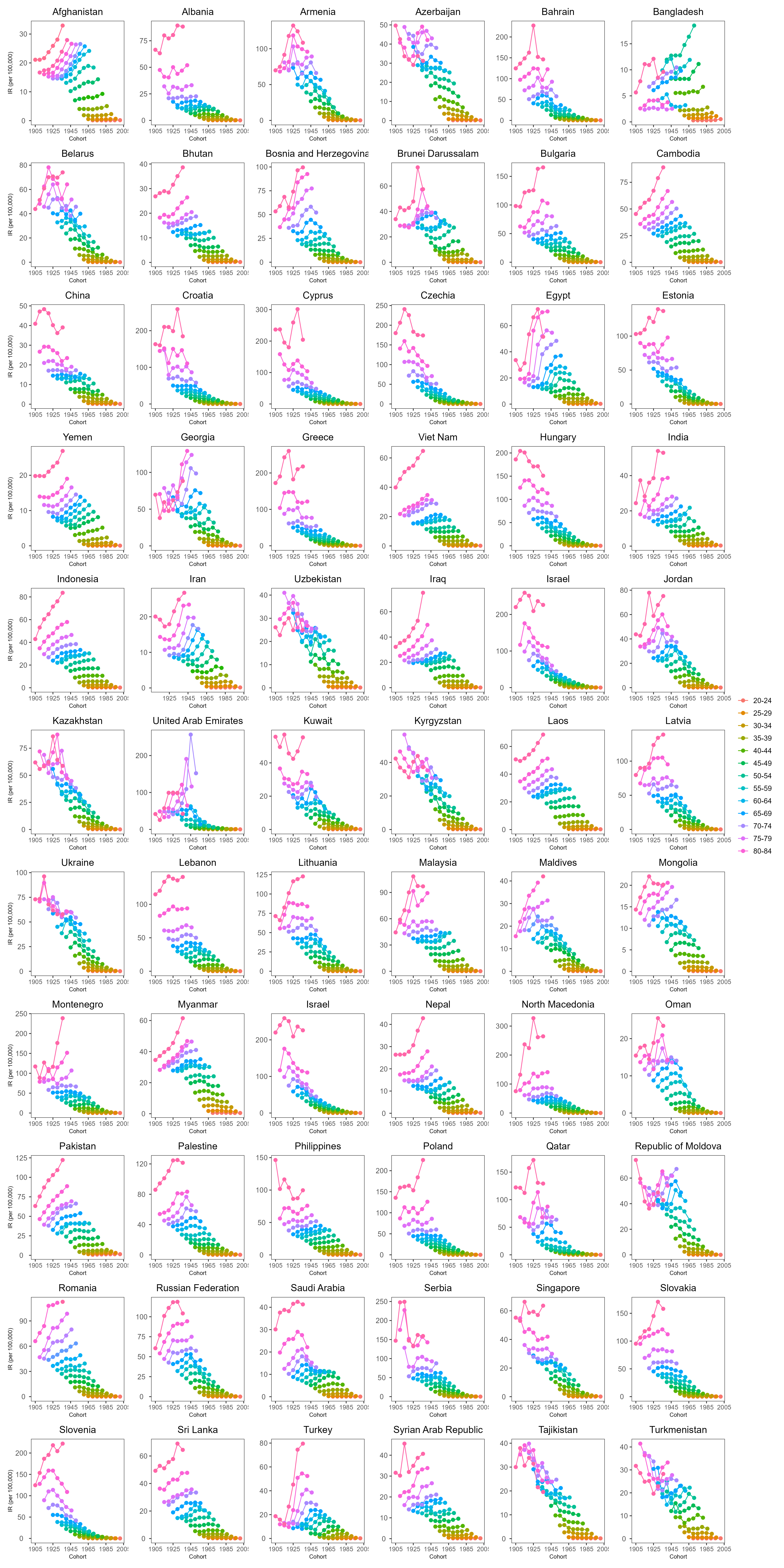

Supplement: Supplementary file 2 — Figure S2: Cohort‐specific mortality rates of breast cancer by age group across 66 BRI countries between 1990 and 2019. BRI, belt and road initiative; MR, mortality rate. [file TCA-16-e70186-s002.tif]

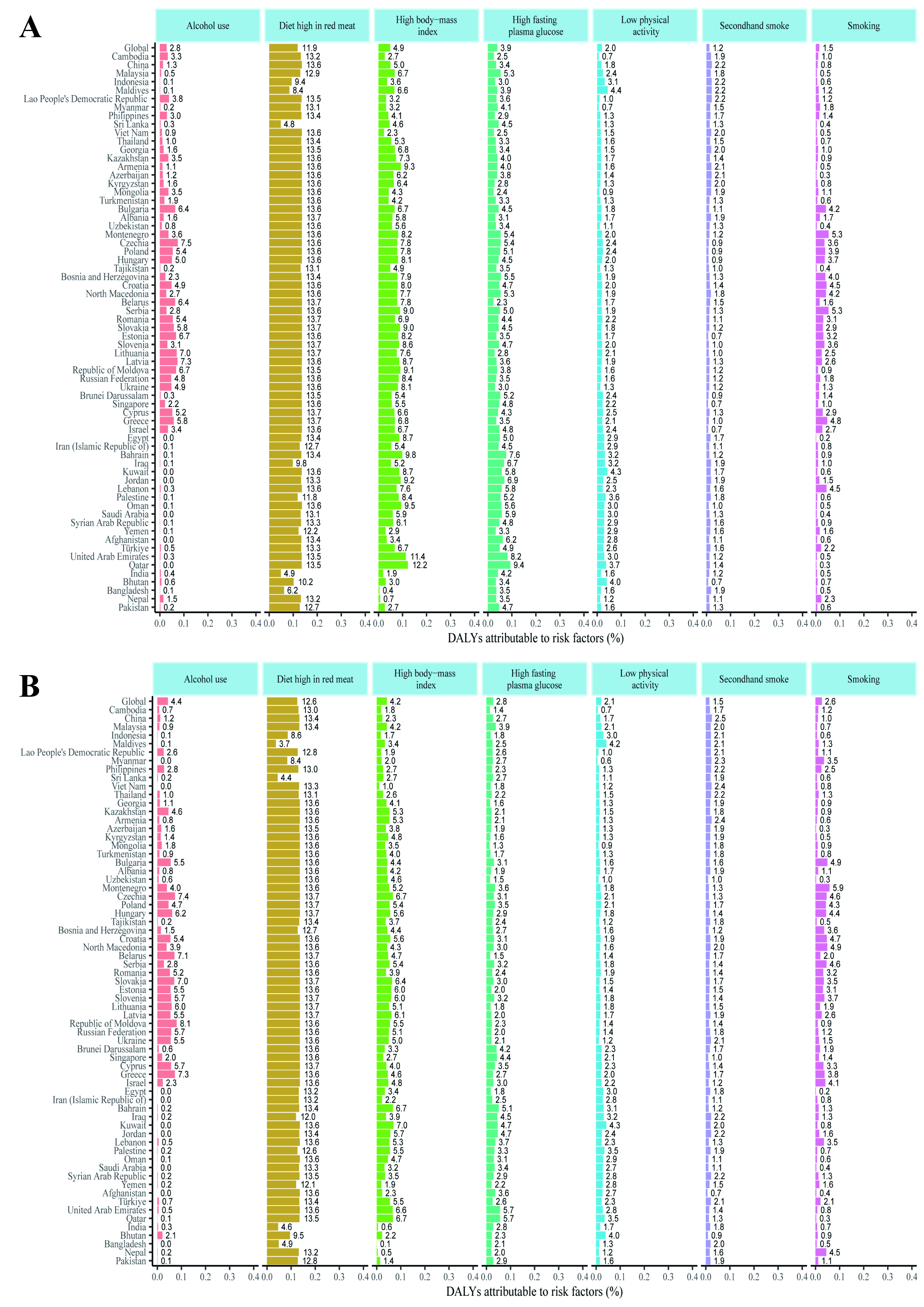

Supplement: Supplementary file 3 — Figure S3: Percentage of DALYs owing to breast cancer attributable to risk factor for 66 BRI countries in 1990 (A) and 2021 (B) in both sexes. BRI, belt and road initiative; DALYs, disability‐adjusted life years. [file TCA-16-e70186-s011.tif]
